# *PDK4* gene positively regulates fat deposition in ovine adipocytes

**DOI:** 10.3389/fnut.2025.1706055

**Published:** 2025-12-12

**Authors:** Cheng Xiao, Shaoying Yang, Wenjun Zhao, Yu Liu, WenWen Fang, Lisheng Miao, Xin Li, Yang Cao, Haiguo Jin, Yang Cao

**Affiliations:** 1 Institute of Animal and Veterinary Sciences, Jilin Academy of Agricultural Sciences, Gongzhuling, China; 2 Research Institute for Farm Animal Biology (FBN), Institute of Muscle Biology and Growth, Dummerstorf, Germany; 3 Institute of Agricultural and Environmental Sciences, Rostock University, Rostock, Germany

**Keywords:** *PDK4* gene, fat deposition, lipid metabolism, *TMEM273* gene, sheep, mice

## Abstract

**Introduction:**

Intramuscular fat (IMF) content is a crucial factor affecting meat quality and flavor in sheep. Our previous studies demonstrated that the *pyruvate dehydrogenase kinase 4* (*PDK4*) expression increases during adipocyte differentiation and is positively correlated with IMF content in sheep. However, the effects of the *PDK4* gene on lipid metabolism in ovine adipocytes remain unclear.

**Methods:**

The effects of *PDK4* overexpression on ovine adipocyte differentiation and fat deposition were investigated. Subsequently, the key lipids and genes affected by *PDK4* overexpression were identified using lipidomic and transcriptomic sequencing. Furthermore, a *PDK4*-knockout NIH/3T3 cell line was generated to verify the evolutionarily conserved functions of *PDK4* and to examine the expression of candidate genes in a murine model.

**Results:**

*PDK4* overexpression significantly increased triglyceride deposition by 2.32-fold in ovine adipocytes (*p* = 0.001) but did not affect PPARγ mRNA or protein expression levels (*p* > 0.05). A total of 80 differentially expressed lipids (DELs) and 24 differentially expressed genes (DEGs) were identified between the overexpression (Over) and negative control (NC) groups, including 20 upregulated DELs, 60 downregulated DELs, 21 upregulated DEGs, and 3 downregulated DEGs. Additionally, *PDK4* overexpression altered lipid content, composition, carbon chain length, and degree of unsaturation in ovine adipocytes. *TMEM273*, one of the DEGs, was negatively affected by *PDK4* overexpression and closely correlated with 7 DELs (adjusted *p* < 0.01, |*R*| > 0.9). In the murine cell model, *PDK4* knockout of NIH/3T3 (KO) cells significantly decreased triglyceride deposition by 0.77-fold (*p* < 0.01) but did not affect *BSCL2* expression (*p* = 0.08). *TMEM273* expression was significantly increased by 2.78-fold in pre-differentiated KO cells (*p* = 0.018) and significantly decreased by 0.83-fold in differentiated KO cells (*p* = 0.005).

**Conclusion:**

*PDK4* positively regulates fat deposition in both ovine and murine adipocytes. *TMEM273* expression is negatively affected by *PDK4* and correlated with lipid metabolism in sheep and mice.

## Introduction

Mutton is increasingly popular among consumers in China due to its nutritional value and flavor. Indigenous sheep breeds in China are characterized by high fecundity, but their low growth rates and inferior meat quality struggle to meet the growing market demand. Dorper and Australian White sheep are renowned for their high growth rates and superior meat quality (1–3). To improve the growth performance and meat quality of indigenous sheep breeds, Dorper or Australian White sheep are commonly crossbred with indigenous breeds to enhance these traits in offspring.

Our previous study investigated the growth performance and meat quality of Dorper × Small-Tailed Han sheep crossbreeds, Australian White × Small-Tailed Han sheep crossbreeds, and Small-Tailed Han sheep under the same feeding conditions, and demonstrated that Dorper × Small-Tailed Han sheep crossbreeds exhibited higher growth rates and better meat quality (4). Our studies found that *pyruvate dehydrogenase kinase 4* (*PDK4*) expression significantly increases during adipocyte differentiation in Small-Tailed Han sheep (5) and is positively correlated with intramuscular fat (IMF) content in Dorper × Small-Tailed Han sheep crossbreeds (4). These results suggest that *PDK4* may be a key regulator of IMF content in sheep.

Meat quality is a vital economic trait in livestock influenced by multiple factors. IMF content is a crucial factor affecting meat quality attributes including, tenderness, juiciness, flavor, and taste (6, 7). Meat with high IMF content brings greater economic value and is highly preferred by consumers in Asia and America (8). IMF content in sheep is mainly regulated by genetic factors and breed under the same feeding conditions (9, 10). Therefore, exploring key genes regulating the formation and accumulation of intramuscular adipocytes is essential for enhancing IMF content and meat quality in sheep.

The pyruvate dehydrogenase complex (PDC) acts as a key metabolic switch and determines glucose or fatty acids oxidation to meet the body’s energy demands. Activated PDC catalyzes the conversion of pyruvate to acetyl-CoA for glucose oxidation, while high cellular ATP concentrations inhibit PDC activity, thereby promoting pyruvate flux toward fatty acid synthesis (11). Pyruvate dehydrogenase kinase 4 (PDK4) phosphorylates and inhibits PDC activity (12). Under high-fat diet conditions, elevated *PDK4* expression inhibits PDC activity and promotes *de novo* fatty acid synthesis (13). Studies showed that *PDK4* expression is increased in pigs with high IMF content and heavy-weight chickens; a single nucleotide polymorphism (SNP) in intron 9 of the *PDK4* gene is closely associated with IMF content in pigs (14, 15). *PDK4* expression is also elevated in the early stages of human obesity (16) and is regulated by plasma free fatty acids, insulin, and rosiglitazone (17). Insulin and rosiglitazone activate the PPARγ and retinoic acid signaling pathways, which in turn influence *PDK4* transcription (18). Several adipogenesis-related transcription factors, including STAT5, KLF15, FOXO1, CIDEA, and PPARGC1A, bind to the *PDK4* promoter to modulate its expression (11, 19–22). *PDK4* not only regulates PDC activity but also modulates calcium ion transfer from the endoplasmic reticulum (ER) to mitochondria by localizing to mitochondria-associated membranes (MAMs) in myoblasts (23, 24). These findings suggest that *PDK4* plays a crucial role in lipid metabolism. However, the effects of *PDK4* adipocyte differentiation and fat deposition in sheep remain unclear.

In this study, we investigated the effects of *PDK4* expression on adipogenesis and explored key lipids and genes affected by *PDK4* in sheep. Our findings may provide novel insights for improving IMF content in sheep.

## Materials and methods

### Animals

Three healthy 2-month-old male lambs (Dorper × Small-Tailed Han crossbred sheep) raised by the Jilin Academy of Agricultural Sciences were randomly selected for preadipocyte isolation. After an overnight fast, the lambs were stunned using a captive bolt pistol, suspended, and exsanguinated. The abdominal skin was incised with a sterile surgical scalpel, and subcutaneous white adipose tissue was aseptically collected from the inguinal region for preadipocyte isolation. The carcasses were then packaged in sealed plastic bags and stored at −20 °C until further processing by trained personnel. All experimental procedures involving animals were approved by the Animal Ethics Committee of the Jilin Academy of Agricultural Sciences (Approval No. JNK 20240530-05).

### Isolation, culture, and differentiation of preadipocytes

The ovine preadipocytes were isolated as described in our previous study (25). Briefly, the adipose tissue was cut into small pieces using sterile scissors and digested with 0.2% collagenase II (Sangon Biotech, Shanghai, China) for 1 h at a 37 °C water bath. The digestate was sequentially filtered through 200- and 400-mesh sieves to remove undigested tissue fragments and large cells. The filtrate was then centrifuged at 500 × g for 5 min to collect the preadipocyte pellet. The cells were cultured in growth medium consisting of Dulbecco’s modified Eagle’s medium (DMEM-F12, Sigma), 10% fetal bovine serum (Gemini Bio-Products, Woodland, CA, United States), and 1% penicillin/streptomycin (Sigma) in a 37 °C, 5% CO₂ incubator. The confluent preadipocytes were differentiated into mature adipocytes using exogenous inducer I and II. First, the cells were treated with inducer I (growth medium supplemented with 10 μg/mL insulin (Sigma), 1.0 μM dexamethasone (Sigma), and 0.5 mM IBMX (Sigma)) for 48 h, followed by treatment with inducer II (growth medium supplemented with 10 μg/mL insulin) for another 48 h. Subsequently, the cells were continuously cultured in growth medium for 4 or 6 days until differentiated into adipocytes.

### Synthesis of overexpression plasmids

The coding sequence (CDS) of the *PDK4* gene (reference sequence: XM_004007738.6) was amplified based on the NCBI reference sequence and cloned into the pEX4 vector using *Xho*I and *Hind*III restriction sites to generate a recombinant overexpression plasmid. The empty pEX4 vector was used as a control. The primer sequences are listed in Supplementary Table 1.

### Transfection and verification

Preadipocytes at 60–70% confluence were transfected with a mixture containing 3 μg of PDK4 overexpression plasmid, 5 μL of Lipofectamine 2,000 (Thermo Fisher Scientific, Waltham, MA, United States), and 200 μL of Opti-MEM (Invitrogen, Carlsbad, CA, United States) per well in a 6-well plate for 6 h. The cells were cultured continuously in growth medium for 48 h. Transfection efficiency was evaluated by quantifying *PDK4* mRNA expression levels using qPCR.

Preadipocytes at 60–70% confluence were transfected with an overexpression plasmid for 24 h, then the cells were treated with inducer I for 48 h (Over group). Preadipocytes without plasmid treatment served as the negative control (NC) group. Subsequently, the lipids and total RNA were extracted from the cells for lipidome and transcriptome sequencing, respectively. Each group included six biological replicates for lipidome sequencing and three for transcriptome sequencing.

### Oil Red O staining and triglyceride quantification

Lipid droplets serve as an indicator of adipocyte maturation because they can be stained red with Oil Red O dye (26). The differentiated adipocytes were fixed with 4% paraformaldehyde (Sangon Biotech, Shanghai, China) for 30 min at room temperature (RT) and washed with PBS three times. The cells were stained with 2% Oil Red O (Sangon Biotech, Shanghai, China) solution at 37 °C for 30 min and then washed again. The images of Oil Red O-stained cells were captured using a microscope. Subsequently, the stained lipid droplets were eluted with isopropanol to measure the absorbance at 490 nm using a microplate reader for quantification. The detailed staining procedure was performed as previously described (25).

Triglyceride content of differentiated adipocytes was measured to evaluate fat deposition using a commercial triglyceride assay kit (Prilax, Beijing, China). The absorbance of triglycerides was measured at 550 nm using a microplate reader, and the concentrations were calculated based on a standard curve as described in the manufacturer’s instructions.

### Lipidome sequencing

The cellular lipids were extracted using the methyl tert-butyl ether (MTBE) method. Briefly, each sample was mixed with 200 μL of water, 240 μL of pre-cooled methanol, and 800 μL of MTBE. The mixture was sonicated at 4 °C for 20 min and incubated at room temperature for 30 min, then centrifuged at 14,000 × g and 10 °C for 15 min. The upper organic phase was collected and evaporated to dryness under a nitrogen stream. Subsequently, the dried lipid extracts were reconstituted in 200 μL of 90% isopropanol/acetonitrile (Thermo Fisher Scientific, Waltham, MA, United States) and centrifuged at 14,000 × g for 15 min. The supernatant was subjected to lipid identification using an LC-MS/MS system.

Chromatographic separation was performed using a reverse-phase CSH C18 column (1.7 μm, 2.1 mm × 100 mm; Waters) on a UHPLC Nexera LC-30A system (Shimadzu, Japan). Mass spectrometry detection was conducted on a Q-Exactive Plus instrument (Thermo Fisher Scientific, Waltham, MA, United States) in both positive and negative electrospray ionization (ESI) modes. The ESI parameters were optimized and maintained consistently across all measurements. The lipids were identified using Lipid Search software based on MS/MS spectral matching, with mass tolerances set to 5 ppm for both precursors and fragments.

Base peak chromatogram (BPC), Pearson correlation analysis, principal component analysis (PCA), and Hotelling’s *T*^2^ test were performed to evaluate the repeatability of lipidomic data. A multivariate control chart (MCC) was used to evaluate the reliability and stability of the data. The differences in lipid concentrations between the two groups were analyzed using Student’s *t*-test. An orthogonal partial least squares-discriminant analysis (OPLS-DA) model was validated by 7-fold cross-validation and 200 permutation tests to minimize noise and identify significant lipids. The model evaluation parameters (*R*^2^*Y* = 0.99, *Q*^2^ = 0.79, and *Q*^2^ intercept = −0.13) confirmed its reliability without overfitting. The lipids with a variable importance in projection (VIP) >1, fold change ≥1.5 or ≤0.67, and *p*-value <0.05 were defined as differentially expressed lipids (DELs). All analyses were conducted using R software.

### Transcriptome sequencing

Total RNA was extracted from cells using TRIzol reagent (Thermo Fisher Scientific, Waltham, MA, United States). RNA degradation and potential contamination were assessed by 1% agarose gel electrophoresis. RNA purity was evaluated using a NanoDrop One spectrophotometer (NanoDrop Technologies, Wilmington, DE, United States), and RNA concentration was measured using a Qubit 3.0 Fluorometer (Life Technologies, Carlsbad, CA, United States). mRNA was isolated using oligo (dT) magnetic beads. The purified mRNA was fragmented and used as a template for synthesizing first- and second-strand cDNA. A cDNA library was constructed through end repair, poly(A) tailing, purification, and PCR amplification. High-quality libraries were sequenced on the DNBSEQ platform (BGI Technologies Inc., Shenzhen, China) by Benagene Gene Technology Co., Ltd. (Wuhan, China).

Low-quality reads and adapter-containing reads were removed from raw reads using fastp (v0.21.0). Quality control of clean reads was performed using FastQC (v0.11.9). Clean reads were mapped to the sheep reference genome (accession number: Oar_rambouillet_v2.0) using HISAT2. Gene expression levels were quantified as fragments per kilobase of transcript per million mapped reads (FPKM). Differential expression analysis was performed using DESeq2 (v1.26.0). Benjamini–Hochberg (BH) procedure was used for multiple testing correction to filter out false positives. Differentially expressed genes (DEGs) were defined as genes with |log₂(fold change)| >1 and adjusted *p*-value (*p*-adj.) <0.05. Gene Ontology (GO) and Kyoto Encyclopedia of Genes and Genomes (KEGG) pathway enrichment analyses were conducted using R software.

### Generation of *PDK4*-knockout NIH/3T3 cell line

A *PDK4*-knockout (KO) NIH/3T3 cell line was established using CRISPR/Cas9-mediated gene editing. Briefly, two guide RNAs (gRNAs) targeting the *PDK4* gene were designed and cloned into the CRISPR-U plasmid. The plasmid was transfected into NIH/3T3 cells seeded in 6-well plates via electroporation (1,300 V, 30 ms, 1 pulse). The cells expressing green fluorescent protein (GFP) were selected using 2 μg/mL puromycin based on viability and fluorescence intensity. A portion of the transfected cells was plated into 96-well plates for single-cell cloning, while genomic DNA was extracted from the remaining cells to assess knockout efficiency by PCR and Sanger sequencing. Single-cell clones were expanded and validated for *PDK4* knockout. Verified knockout cells were cultured and cryopreserved for subsequent experiments. Wild-type (WT) NIH/3T3 cells served as the control. The sequences of gRNAs and related primers are listed in Supplementary Table 1, and validation results are provided in Supplementary Figure 1.

### RNA extraction and qPCR verification

Total RNA was extracted from adipocytes and NIH/3T3 cells using TRIzol reagent (Thermo Fisher Scientific, Waltham, MA, United States). RNA quality and concentration were determined using a NanoDrop spectrophotometer (Thermo Fisher Scientific, Waltham, MA, United States). Complementary DNA (cDNA) was synthesized using a reverse transcription kit (TaKaRa, Shiga, Japan) following the manufacturer’s instructions. Relative gene expression was quantified using a Roche LightCycler^®^ 480 Real-Time PCR System (Roche Applied Science, Penzberg, Germany) as previously described protocol (25). Glyceraldehyde-3-phosphate dehydrogenase (*GAPDH*) was used as the housekeeping gene. The relative gene expression levels were statistically analyzed using the 2^−ΔΔCt^ method. The primer sequences are listed in Supplementary Table 1.

### Western blotting

Total protein was extracted from adipocytes using a commercial protein extraction kit (Solarbio, Shanghai, China) according to the manufacturer’s instructions. Protein concentration was determined using a BCA assay kit (Beyotime, Jiangsu, China). Subsequently, the proteins of all samples were diluted with lysis buffer to achieve uniform concentrations. Western blot analysis was performed as previously described (25). Primary antibodies against peroxisome proliferator-activated receptor *γ* (PPARG, bs-0530R, Bioss, Beijing, China) and β-actin (bs-1571R, Bioss, Beijing, China) were diluted at ratios of 1:1,000 and 1:5,000, respectively. Protein bands were visualized using a ChemiScope 6,000 Touch imaging system (Clinx Science Instruments, Shanghai, China) and quantified with ImageJ software (National Institutes of Health, Bethesda, MD, United States).

### Statistical analysis

The data are presented as mean ± SEM. Comparisons between two groups or among multiple groups were performed using Student’s *t*-test or one-way analysis of variance (ANOVA) with Tukey’s honest significant difference (HSD) *post hoc* test, respectively, using SPSS 17.0 software (IBM, Armonk, NY, United States). Figures were drawn using GraphPad Prism 6.0 (GraphPad Software, San Diego, CA, United States). A *p*-value <0.05 was deemed statistically significant.

Joint lipidome and transcriptome analysis, two-way orthogonal partial least squares (O2PLS), canonical correlation analysis (CCA), and Pearson correlation analysis were conducted using R software to identify the key variables and calculate their correlations. First, O2PLS, an unsupervised model, was used to evaluate the impact and weights of different variables; variables with higher weights exert greater influence on others; Lipids or genes with an O2PLS weight >0.2 were defined as important variables. CCA was performed to identify and quantify intercorrelations within and between differentially expressed lipids (DELs) and differentially expressed genes (DEGs) by identifying linear combinations; a correlation coefficient close to 1 indicates a strong positive correlation between variables. Pearson correlation analysis was used to calculate correlation coefficients between univariate variables. Variables with a correlation coefficient >0.9 were deemed key variables. Original images and raw data are provided in Supplementary material.

## Results

### *PDK4* overexpression promotes triglyceride deposition in ovine adipocytes

**Figure 1:**
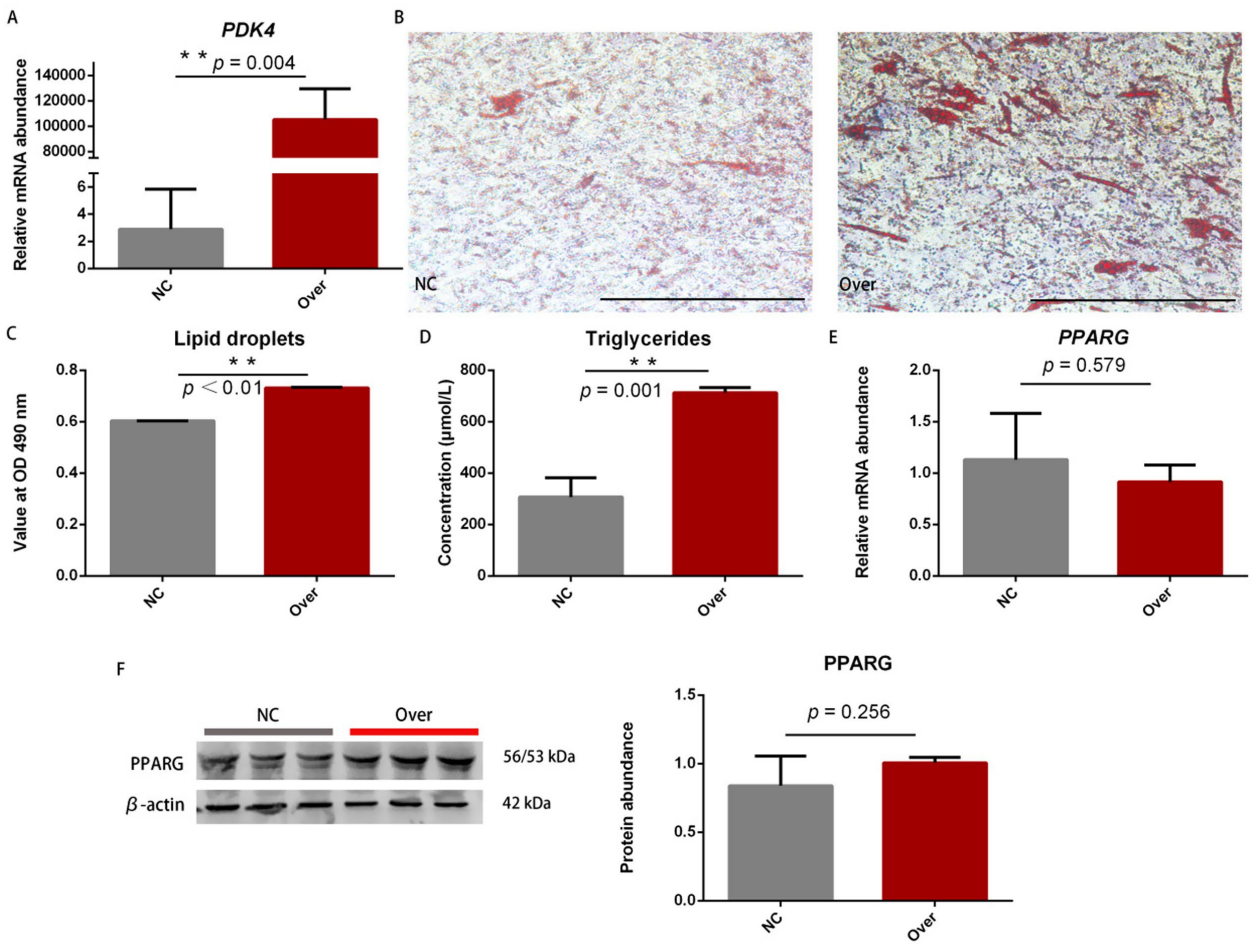
Effects of *PDK4* on ovine adipocyte differentiation and triglyceride deposition. **(A)** *PDK4* expression levels between the overexpression (Over) and negative control (NC) groups (*p* = 0.004). **(B)** Oil Red O staining of adipocytes between the two groups. Red deposits indicate lipid droplets. Scale bar: 100 μm. **(C)** Quantification of lipid droplets assessed by absorbance at 490 nm (*p* < 0.01). **(D)** Triglyceride concentration in adipocytes between the two groups (*p* = 0.001). **(E)** PPARγ mRNA expression levels between the two groups (*p* = 0.579). **(F)** PPARγ protein expression levels between the two groups (*p* = 0.256). ^*^p < 0.05 and ^**^p < 0.01.

The effects of *PDK4* gene expression on adipocyte differentiation and fat deposition were investigated in sheep. The overexpression plasmid significantly increased *PDK4* mRNA levels by 36,343-fold compared to the negative control (NC) group (*p* = 0.004; Figure 1A). Oil Red O staining images of the overexpression (Over) and control (NC) groups are shown in Figure 1B. Quantification of lipid droplets revealed that the absorbance of lipid droplets in the Over group was 1.21-fold higher than that in the NC group (*p* < 0.01; Figure 1C). Additionally, *PDK4* overexpression significantly increased the content of triglyceride by 2.32-fold compared to the NC group (*p* = 0.001; Figure 1D).

PPARγ is a well-recognized biomarker of adipocyte differentiation across multiple species, including humans, rodents, ruminants, fish, and poultry (27–30). In the current study, there were no significant differences in PPARγ mRNA and protein expression levels between the Over and NC groups (*p* > 0.05; Figures 1E,F). These results indicate that *PDK4* overexpression promotes triglyceride deposition in ovine adipocytes but does not affect adipocyte differentiation biomarker PPARγ. Raw data are provided in Supplementary Table 2.

### Lipidome analysis

**Figure 2:**
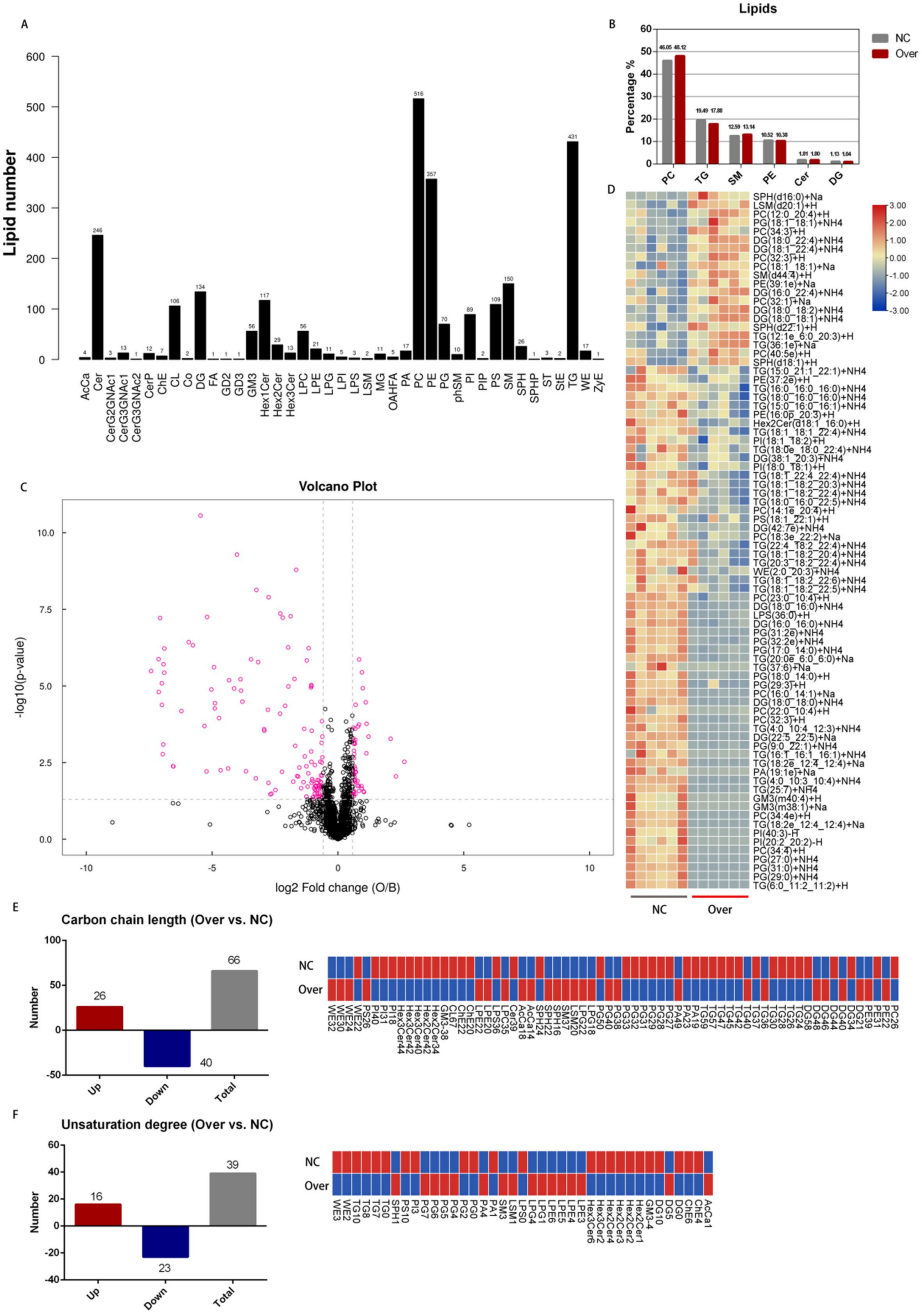
Lipidomic profiles between the over and control group. **(A)** Number and categories of lipids identified in lipidomic profiles. **(B)** Proportional distribution of major lipid classes in the Over and NC groups. **(C)** Volcano plot of differentially expressed lipids (DELs), pink bubbles on the left represent downregulated DELs, the bubbles on the right represent upregulated DELs. **(D)** Heatmap of DELs (VIP >1, *p* < 0.05). **(E)** Number (left) and species (right) of lipids with different carbon chain lengths; red represents upregulated lipids and blue represents downregulated lipids. **(F)** Number (left) and species (right) of lipids with different degrees of unsaturation.

The effects of *PDK4* gene expression on lipid profiles in adipocytes were investigated via lipidome analysis. Quality control analysis demonstrated that the lipidomic data exhibited high quality, stability, and reproducibility (Supplementary Figure 2). A total of 2,661 lipid species belonging to 41 classes were identified (Figure 2A). The proportions of phosphatidylcholine (PC), triglyceride (TG), sphingomyelin (SM), phosphatidylethanolamine (PE), ceramide (Cer), and diglyceride (DG) between the two groups are shown in Figure 2B. Among lipid classes, the concentrations of lipopolysaccharide (LPS), phosphatidylglycerol (PG), dihexosylceramide (Hex₂Cer), and trihexosylceramide (Hex₃Cer) were higher in the NC group (*p* < 0.05), while lysosphingomyelin (LSM) and lysophosphatidylglycerol (LPG) were higher in the Over group (*p* < 0.05) (Table 1). At the lipid species, 80 differentially expressed lipids (DELs) were identified between the Over and NC groups, including 20 upregulated and 60 downregulated lipids (Figures 2C,D and Supplementary Table 3).

**Table 1:** Instrument response value^a^ of lipid classes between the two groups.

Lipid class	Negative control		Overexpression		
	Mean	SEM	Mean	SEM	*p*-value
LPS	1.79 × 10^7^	1.23 × 10^6^	6.87 × 10^6^	5.06 × 10^5^	0
PG	1.67 × 10^9^	9.25 × 10^7^	1.04 × 10^9^	4.87 × 10^7^	0
Hex_2_Cer	8.10 × 10^7^	2.28 × 10^6^	6.76 × 10^7^	2.12 × 10^7^	0.001
Hex_3_Cer	2.64 × 10^7^	1.68 × 10^6^	2.04 × 10^7^	9.53 × 10^5^	0.011
LSM	9.89 × 10^6^	4.49 × 10^6^	3.71 × 10^7^	3.97 × 10^6^	0.001
LPG	6.71 × 10^6^	5.06 × 10^5^	8.55 × 10^6^	6.01 × 10^5^	0.042

a Instrument response with higher value indicates higher lipid concentration.

Lipid carbon chain length and degree of unsaturation influence membrane fluidity, signal transduction, and cellular physiological status (31, 32). With respect to carbon chain length, there were 66 lipids with significantly different concentrations between the two groups, of which 26 had higher concentrations in the Over group and 40 had higher concentrations in the NC group (*p* < 0.05; Figure 2E). Regarding the degree of unsaturation, there were 39 lipids with significantly different concentrations between the two groups, of which 16 had higher concentrations in the Over group and 23 had higher concentrations in the NC group (*p* < 0.05; Figure 2F). Raw data are provided in Supplementary Tables 4, 5. Taken together, *PDK4* overexpression alters the lipid content, composition, carbon chain length, and degree of unsaturation in ovine adipocytes.

### Transcriptome analysis

**Figure 3:**
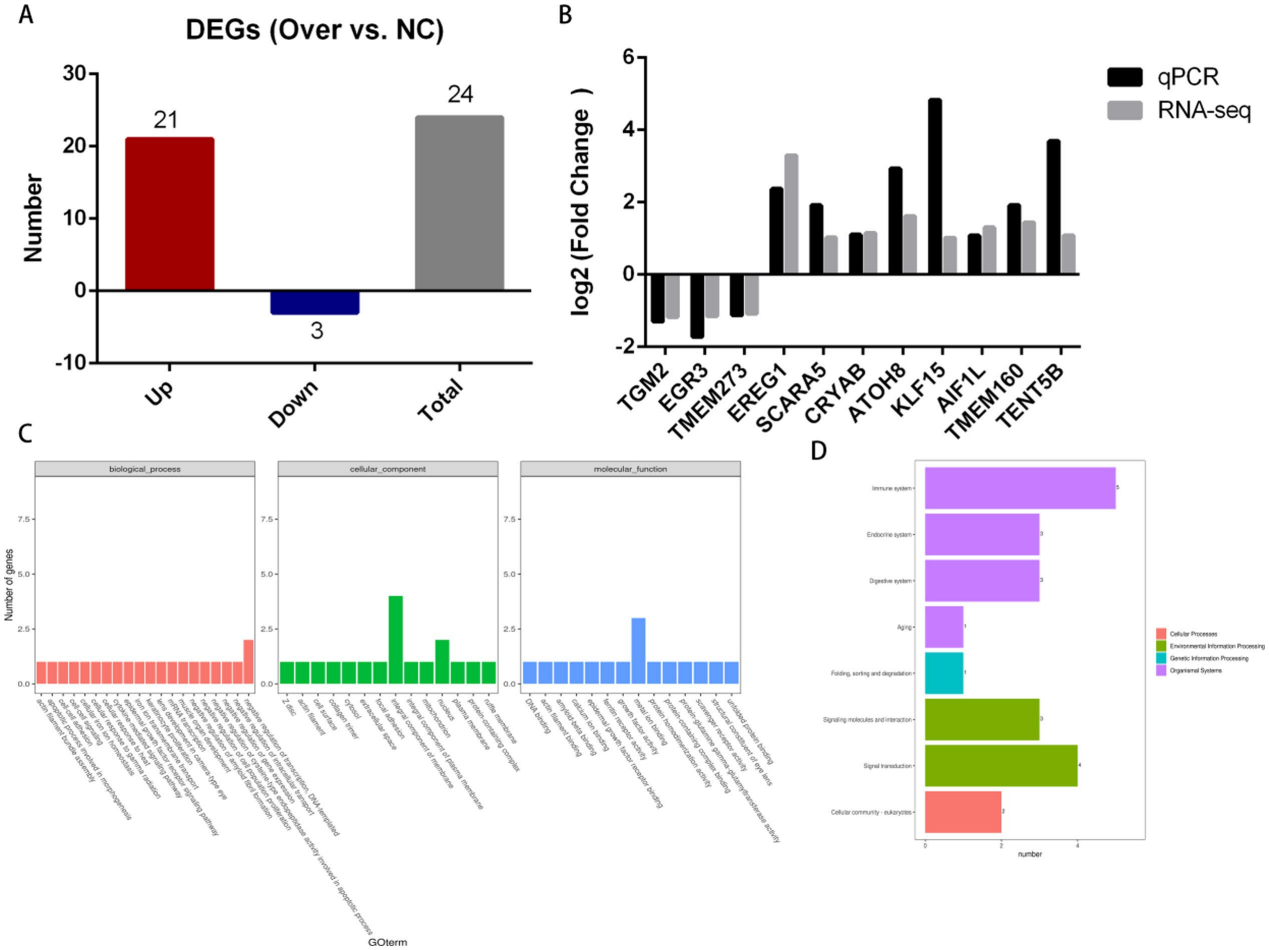
Transcriptome analysis between the Over and NC groups. **(A)** Number of differentially expressed genes (DEGs) between the two groups. **(B)** Comparison of qPCR results and RNA-seq results for selected DEGs. **(C)** Gene Ontology (GO) enrichment analysis of DEGs **(D)** Kyoto Encyclopedia of Genes and Genomes (KEGG) enrichment analysis of DEGs.

*PDK4* regulates pyruvate dehydrogenase complex activity or facilitates Ca^2+^ transfer from the endoplasmic reticulum (ER) to mitochondria via mitochondria-associated membranes (MAMs) to modulate cellular physiological processes (24, 33). To identify downstream genes regulated by *PDK4*, transcriptome analysis was performed. The sequencing data exhibited high quality, stability, and reproducibility, with sample mapping rates exceeding 92%. A total of 24 differentially expressed genes (DEGs) were identified between the Over and NC groups, including 21 upregulated and 3 downregulated DEGs (Figure 3A and Table 2).

**Table 2:** Differentially expressed genes (DEGs) between the Over and NC groups.

Genes	Log2 (FoldChange)	*p*-value	*p*-adj.	Diff. type
LOC121818524	4.31	2.08 × 10^−205^	6.01 × 10^−202^	Up
LOC114112700	3.66	3.11 × 10^−58^	4.00 × 10^−55^	Up
novel170	3.57	1.15 × 10^−52^	1.33 × 10^−49^	Up
novel137	3.35	4.16 × 10^−122^	8.02 × 10^−119^	Up
novel171	3.34	0	0	Up
novel962	3.32	8.69 × 10^−83^	1.26 × 10^−79^	Up
novel172	3.30	9.60 × 10^−172^	2.22 × 10^−168^	Up
EREG	3.29	9.69 × 10^−28^	9.34 × 10^−25^	Up
novel138	3.29	1.47 × 10^−91^	2.42 × 10^−88^	Up
novel961	3.08	0	0	Up
novel169	2.30	5.41 × 10^−245^	2.09 × 10^−241^	Up
ATOH8	1.61	2.83 × 10^−08^	4.55 × 10^−6^	Up
TMEM160	1.44	9.24 × 10^−06^	4.97 × 10^−4^	Up
AIF1L	1.30	3.97 × 10^−10^	9.57 × 10^−8^	Up
CRYAB	1.15	1.02 × 10^−4^	3.43 × 10^−3^	Up
novel142	1.13	2.13 × 10^−4^	6.02 × 10^−3^	Up
TENT5B	1.09	7.30 × 10^−7^	6.65 × 10^−5^	Up
novel559	1.07	1.94 × 10^−3^	0.03	Up
LOC101114226	1.07	2.77 × 10^−5^	0.0012	Up
SCARA5	1.03	7.04 × 10^−6^	4.05 × 10^−4^	Up
KLF15	1.02	4.92 × 10^−6^	3.03 × 10^−4^	Up
TMEM273	−1.08	1.44 × 10^−6^	1.12 × 10^−4^	Down
EGR3	−1.15	2.57 × 10^−7^	2.80 × 10^−5^	Down
TGM2	−1.17	2.00 × 10^−14^	1.29 × 10^−11^	Down

*TGM2*, *EGR3*, *TMEM273*, *SCARA5*, *TENT5B*, *CRYAB*, *AIF1L*, *ATOH8*, *EREG*, *KLF15*, and *TMEM160* were selected to validate the accuracy and reliability of RNA-seq via qPCR. The results demonstrated that RNA-seq was accurate and reliable, as the expression trends of these genes were consistent with the sequencing data (Figure 3B; raw data provided in Supplementary Table 6).

Gene Ontology (GO) enrichment analysis indicated that *TMEM273*, *TMEM160*, *EREG*, and *LOC101114226* were enriched in the “integral component of the membrane” term; *EGR3* and *CRYAB* were enriched in “nucleus”; *EGR3*, *TGM2*, and *CRYAB* were enriched in “metal ion binding”; *EREG* and *CRYAB* were enriched in “negative regulation of transcription, DNA-templated” (Figure 3C). Kyoto Encyclopedia of Genes and Genomes (KEGG) enrichment analysis revealed that the DEGs were primarily enriched in the immune system, signal transduction, endocrine system, digestive system, and signaling molecules and interaction pathways (Figure 3D).

### Joint analysis of lipidome and transcriptome

**Figure 4:**
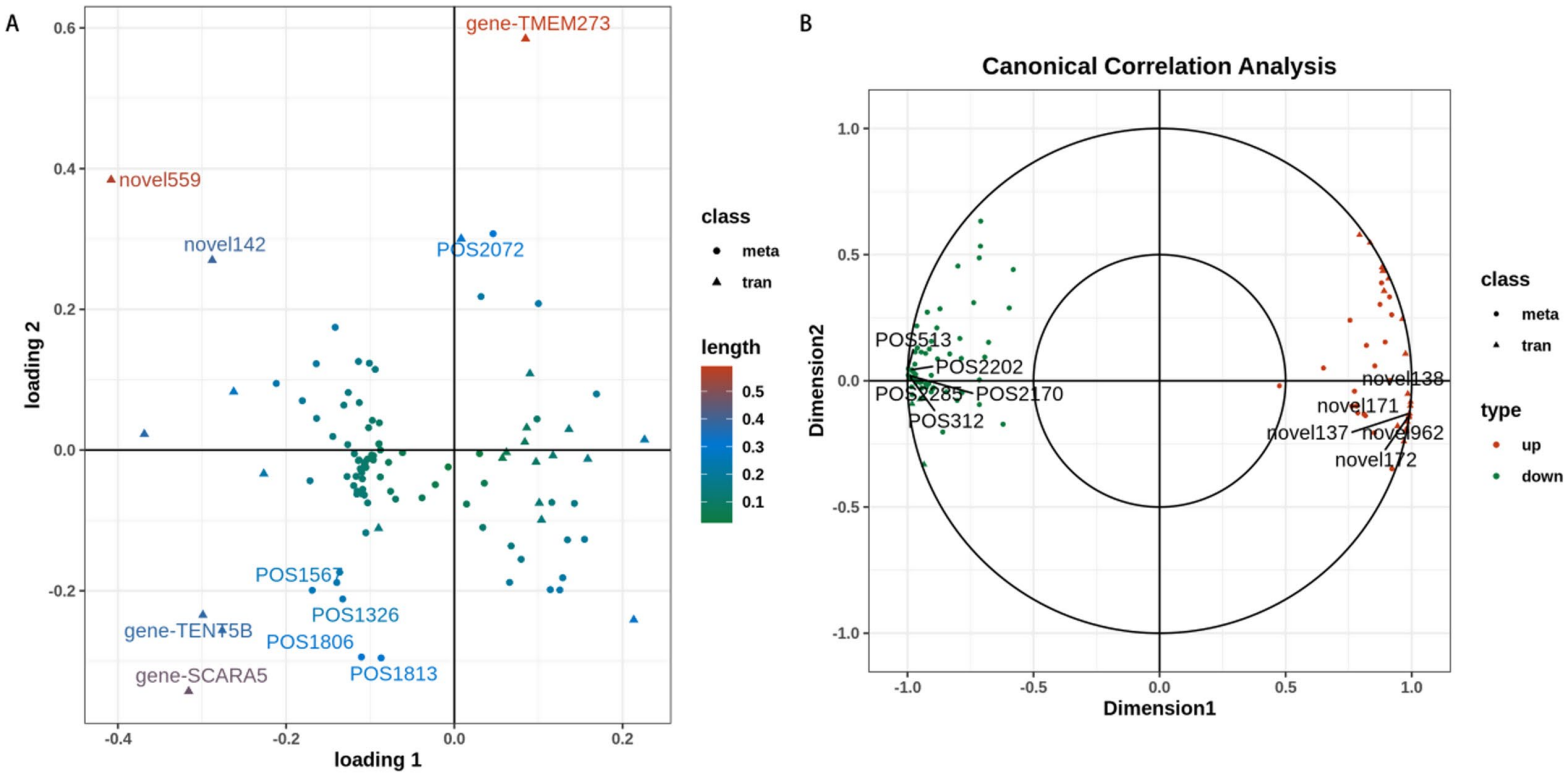
Joint analysis of transcriptomic and lipidomic profiles. **(A)** Two-way orthogonal partial least squares (O2PLS) score plot of DEGs and differentially expressed lipids (DELs); variables farther from the origin exert a more significant impact on other variables. **(B)** Canonical correlation analysis (CCA) plot of DEGs and DELs; the closer the distance between variables, the stronger their correlation.

A two-way orthogonal partial least squares (O2PLS) model identified 29 variables with a score >0.2, including 12 differentially expressed genes (DEGs) and 17 differentially expressed lipids (DELs). These variables exerted stronger influences on other variables, among which TMEM273 had the highest score (Figure 4A and Table 3). Canonical correlation analysis (CCA) demonstrated that the scores of the 12 DEGs were all >0.95, while TG (16:0_16:0_16:0) + NH_4_, TG(18:0_16:0_16:0) + NH_4_, and Hex_2_Cer(d18:1_16:0) + H had scores >0.9 (Figure 4B and Table 3).

**Table 3:** The score of O2PLS and CCA of DEGs and DELs.

Genes	O2PLS	CCA	Type	Lipids	Lipids ID	O2PLS	CCA	Type
TMEM273	0.591	0.954	Down	PI(18:0_18:1) + H	POS1806	0.314	0.663	Down
novel559	0.560	0.981	Up	SPH(d16:0) + Na	POS2072	0.311	0.652	Up
SCARA5	0.466	0.99	Up	PI(18:1_18:2) + H	POS1813	0.308	0.695	Down
novel142	0.395	0.959	Up	PE(37:2e) + H	POS1567	0.261	0.865	Down
TENT5B	0.380	0.995	Up	PC(18:3e_22:2) + Na	POS1326	0.250	0.800	Down
ATOH8	0.377	0.995	Up	PG(18:1_18:1) + NH_4_	POS1769	0.235	0.821	Up
CRYAB	0.369	0.998	Up	PS(18:1_22:1) + H	POS1903	0.235	0.729	Down
TMEM160	0.322	0.961	Up	TG(16:0_16:0_16:0) + NH_4_	POS2351	0.232	0.951	Down
novel961	0.301	0.986	Up	SPH(d22:1) + H	POS2124	0.231	0.828	Up
LOC101114226	0.275	0.987	Up	DG(18:0_18:1) + NH_4_	POS361	0.229	0.774	Up
AIF1L	0.229	0.994	Up	TG (18:1_22:4_22:4) + NH_4_	POS2908	0.225	0.810	Down
EGR3	0.227	0.991	Down	TG(12:1e_6:0_20:3) + H	POS2252	0.223	0.788	Up
				TG(36:1e) + Na	POS2225	0.223	0.788	Up
				Hex_2_Cer(d18:1_16:0) + H	POS612	0.221	0.908	Down
				PC(34:3) + H	POS1060	0.220	0.792	Up
				TG(18:0_16:0_16:0) + NH_4_	POS2396	0.205	0.917	Down
				DG(18:0_18:2) + NH_4_	POS364	0.200	0.876	Up

Pearson correlation analysis was performed to assess the relationships between these DEGs and DELs. The results showed that the *TMEM273* was positively correlated with TG(18:0_16:0_16:0) + NH_4_ and TG(18:1_22:4_22:4) + NH_4_, and negatively correlated with DG(18:0_18:2) + NH_4_, TG(36:1e) + Na, TG(12:1e_6:0_20:3) + H, DG(18:0_18:1) + NH_4_, and PG(18:1_18:1) + NH_4_. *TMEM160* was negatively correlated with TG(18:0_16:0_16:0) + NH_4_ (Table 4). The correlation coefficients of other DEGs and DELs were all below 0.9 or above −0.9 (Supplementary Table 7). Taken together, *TMEM273* was closely correlated to 7 DELs and may be a key factor affecting lipid metabolism in ovine adipocytes.

**Table 4:** The correlation coefficients of DEGs and DELs.

Correlation coefficients	TG (18:0_16:0_16:0) + NH_4_	TG (18:1_22:4_22:4) + NH_4_	DG (18:0_18:2) + NH_4_	TG (36:1e) + Na	TG (12:1e_6:0_20:3) + H	DG (18:0_18:1) + NH_4_	PG (18:1_18:1) + NH_4_
TMEM273	0.922	0.917	−0.939	−0.921	−0.921	−0.92	−0.912
TMEM160	−0.903	−0.813	0.888	0.812	0.813	0.806	0.860

### Effects of *PDK4* on NIH/3T3 cell differentiation and fat deposition

**Figure 5:**
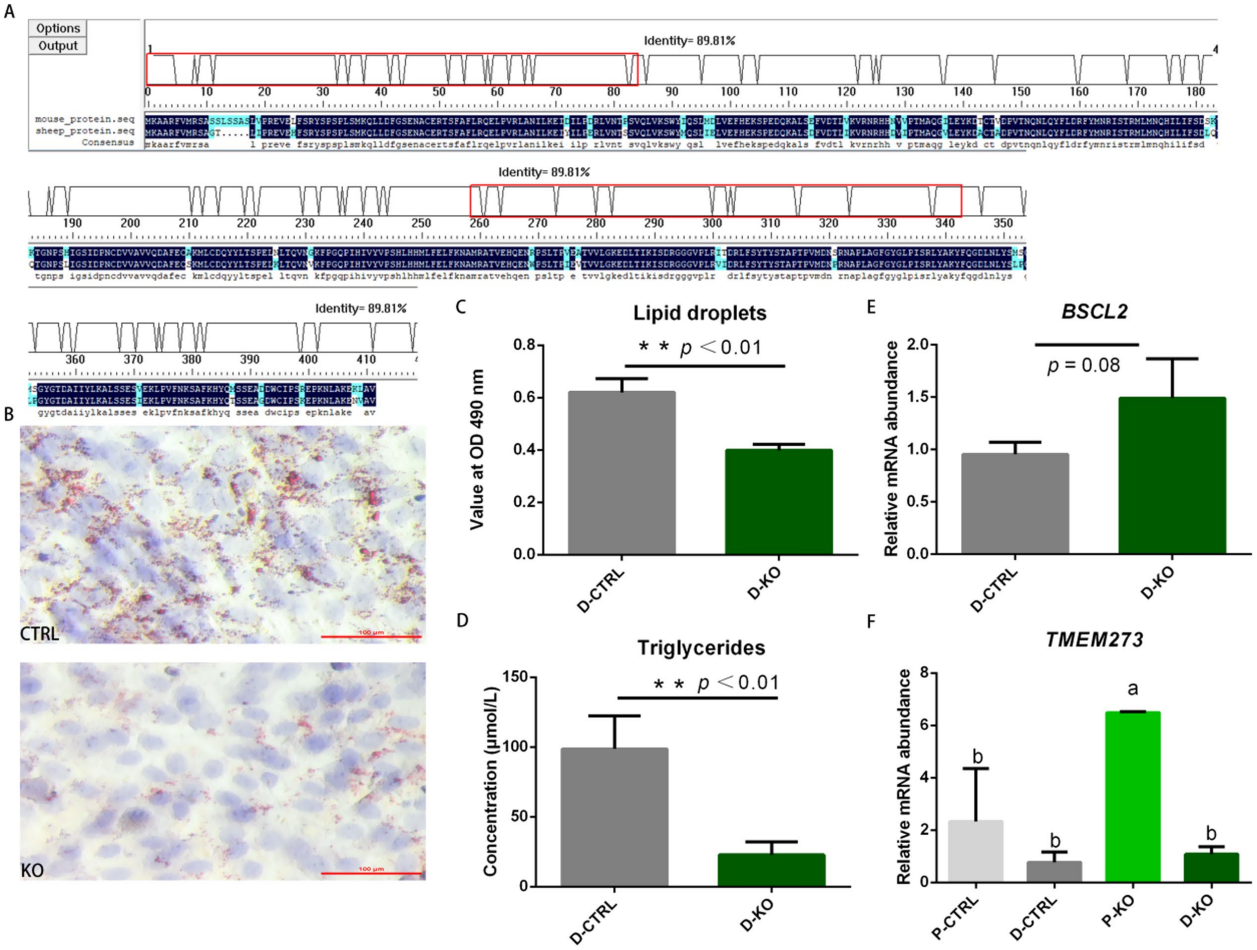
Effects of PDK4 on NIH/3T3 cell differentiation and fat deposition. **(A)** Amino acid sequence alignment of the *PDK4* coding sequence (CDS) region. Line 1: mouse (*PDK4*, NM_013743.2); Line 2: sheep (*PDK4*, XM_004007738.6); Line 3: conserved regions. **(B)** Oil Red O staining of lipid droplets (red) and nuclei (blue) in control (CTRL) and *PDK4*-knockout (KO) cells. Scale bar: 100 μm. **(C)** Quantification of lipid droplets by absorbance at 490 nm in differentiated CTRL and KO cells. **(D)** Triglyceride concentration in differentiated CTRL and KO cells. **(E)** *BSCL2* expression in differentiated CTRL and KO cells. **(F)** *TMEM273* expression in pre-differentiated and differentiated CTRL and KO cells. Different letters indicate significant differences; *p* = 0.018 (P-KO vs. P-CTRL); *p* = 0.003 (P-KO vs. D-CTRL); *p* = 0.005 (P-KO vs. D-KO). ^*^p < 0.05 and ^**^p < 0.01.

The amino acid sequence of PDK4 is conserved between sheep and mice, with 89.81% similarity (15) (Figure 5A). *PDK4*-knockout (KO) NIH/3T3 cells exhibited lower content of lipid droplet (0.64-fold) and triglyceride (0.23-fold) compared to wild-type controls (CTRL) during adipocyte differentiation (*p* < 0.01; Figures 5B–D). *BSCL2* is a key gene required for lipid droplet formation (34). However, no significant difference in *BSCL2* expression was observed between the differentiated KO and CTRL group (*p* = 0.08; Figure 5E).

*TMEM273* expression was significantly increased (2.78-fold) in pre-differentiated KO cells compared to pre-differentiated CTRL cells (*p* = 0.018), and significantly decreased (0.83-fold) in differentiated KO cells (*p* = 0.005; Figure 5F). Raw data are provided in Supplementary Table 8.

These results indicate that *PDK4* knockout suppresses triglyceride deposition in NIH/3T3 cells without altering *BSCL2* expression. Additionally, *TMEM273* expression was increased in *PDK4*-knockout NIH/3T3 cells and decreased during adipocyte differentiation.

## Discussion

Small-Tailed Han sheep are an indigenous breed widely raised in China for their high reproductive performance and strong environmental adaptability, but their meat yield and quality do not meet current market demands (35). Therefore, Dorper rams are commonly crossbred with Small-Tailed Han ewes to improve meat yield and quality in offspring. However, the meat traits of crossbred offspring remain inferior to those of purebred Dorper sheep. Molecular breeding has become a key approach to enhancing the meat traits of the offspring.

Intramuscular fat (IMF) content plays a critical role in meat quality attributes such as flavor, tenderness, juiciness, and nutritional value (36). IMF content exhibits low to moderate heritability and is influenced by both genetic and dietary factors (9). Genetic factors are the primary determinant of IMF content under consistent feeding conditions. Our previous studies demonstrated that *PDK4* expression is significantly upregulated during adipocyte differentiation and is positively correlated with IMF content in Dorper × Small-Tailed Han crossbred sheep (4, 5). However, the effects of *PDK4* expression on ovine adipocyte differentiation and triglyceride deposition, as well as the key lipids and genes influenced by *PDK4*, remain to be fully elucidated.

Adipocyte maturation is roughly divided into two key processes: adipocyte differentiation and triglyceride deposition (37). Adipocyte differentiation: peroxisome proliferator-activated receptor *γ* (PPARγ) initiates the differentiation of preadipocytes into adipocytes (38). Triglyceride deposition: *de novo* synthesized or exogenous fatty acids are esterified into triglycerides, which leads to adipocyte hypertrophy (39). *Pyruvate dehydrogenase kinase 4* (*PDK4*) plays a crucial role in regulating pyruvate metabolic flux. Under energy-deficient conditions, upregulated *PDK4* inhibits pyruvate dehydrogenase complex (PDC) activity, reduces glucose oxidation, and promotes fatty acid oxidation. Under energy-sufficient conditions, *PDK4* expression is also upregulated and suppresses glucose oxidation but promotes pyruvate flux toward *de novo* fatty acid synthesis (11–13, 40). Thus, both cellular energy status and *PDK4* expression levels determine the metabolic fate of pyruvate. During adipocyte differentiation, cells possess sufficient energy to support maturation. Therefore, *PDK4* overexpression promoted triglyceride deposition in ovine adipocytes, which is consistent with the known regulatory mechanism of *PDK4*. Also, in the murine cell model, *PDK4*-knockout NIH/3T3 cells had lower content of triglyceride.

PPARγ is a well-established biomarker that determines the success of adipocyte differentiation (38). No significant difference in PPARγ expression was observed in this study, indicating that *PDK4* regulates fat deposition but does not affect PPARγ expression. PPARγ initiates adipocyte differentiation by activating downstream transcription factors and genes such as the lipid droplet formation marker gene *BSCL2* (38, 41). Consistent with the PPARγ results, no significant difference in *BSCL2* expression was detected in *PDK4*-knockout NIH/3T3 cells. These results suggest that *PDK4* may not influence PPARγ-mediated adipocyte differentiation. More evidence is required to confirm this conclusion in further experiments.

Lipidomic analysis was performed to identify lipids affected by *PDK4* overexpression in ovine adipocytes. Phosphatidylcholine (PC) and phosphatidylethanolamine (PE) are fundamental components of organelle membranes, including the monolayer of lipid droplets and the bilayers of mitochondria and the endoplasmic reticulum (ER) (24). Triglycerides (TG) are the primary storage form of esterified fatty acids in adipocytes, while diglycerides (DG) serve as substrates for triglyceride synthesis (40). Sphingomyelins (SM) are structural lipids that regulate signal transduction, and Ceramides (Cer) are important signaling lipids that reflect cellular physiological status (24). These lipid classes are ubiquitous and critical for cellular and organ function. Thus, the majority of lipids identified in this study belonged to these classes.

Lysophosphatidylserines (LPS) are metabolites of phosphatidylserine (PS) and signaling molecules that regulate immune and inflammatory responses (42). Phosphatidylglycerols (PG) are key components of organelle membranes (24). Lysophosphatidylglycerols (LPG) are metabolites of PG and signaling molecules that regulate intracellular calcium transport to influence triglyceride accumulation (43, 44). Dihexosylceramides (Hex_2_Cer) and trihexosylceramides (Hex_3_Cer) belong to glycosphingolipids and play crucial roles in signal transduction and lipid raft formation (45). Lysosphingomyelins (LSM) are metabolites of SM and important signaling molecules that affect cell proliferation, differentiation, and apoptosis (46). The concentrations of these lipid classes were influenced by *PDK4* overexpression in this study, indicating that they may be involved in *PDK4* overexpression regulating fat deposition in ovine adipocytes.

A previous study identified PC (16:0/18:3) as a major lipid component in mutton (47). In the present study, the concentration of PC (16:0/18:3), one of differentially expressed lipids (DELs), also increased in the overexpression (Over) group. Lipid carbon chain length and degree of unsaturation influence membrane fluidity, signal transduction, and metabolic regulation (24). A total of 66 lipids showed significant differences in carbon chain length, and 39 lipids showed significant differences in degree of unsaturation between the two groups in this study. These results demonstrate that *PDK4* overexpression influences lipid content, composition, carbon chain length, and degree of unsaturation in ovine adipocytes.

Transcriptomic sequencing identified 24 differentially expressed genes (DEGs) in this study. Eleven DEGs with functional annotations were selected by qPCR validation to confirm the reliability of RNA-seq data. The expression trends of these DEGs were consistent with those from RNA-seq, indicating that the RNA-seq results are reliable. Transglutaminase 2 (*TGM2*) localizes to the cell surface, cytosol, and organelle membranes, and is expressed in various tissues (e.g., white adipose tissue). It plays a crucial role in cell proliferation and differentiation (48). A study demonstrated that *TGM2* acts as an adipogenesis inhibitor that suppresses adipocyte differentiation (48). Consistent with this, *TGM2* expression was reduced in the *PDK4* overexpression (Over) group in this study, supporting its role as a negative regulator of fat deposition. The Early growth response (EGR) family consists of four zinc-finger transcription factors (EGR1–4). EGR1 and EGR2 exert opposing effects on adipogenesis (49), while the role of EGR3 remains poorly characterized. A study showed that *EGR3* expression was higher in Iberian pigs with low intramuscular fat (IMF) content (50). In the present study, *EGR3* expression was decreased in the Over group, which aligns with this report. Obesity-induced inflammation promotes the secretion of pro-inflammatory molecules and adipocyte degradation via macrophages, with allograft inflammatory factor 1-like (*AIF1L*) being involved in this process (51). *AIF1L* is associated with obesity and dynamically expressed during adipogenesis in goats (52). Consistent with these findings, *AIF1L* expression was increased in the Over group in the present study. The Krüppel-like factor (KLF) family regulates lipid metabolism and adipocyte differentiation. *KLF15*, a member of this family, has been shown to promote fat deposition (53). In this study, *KLF15* expression was also increased in the Over group. Alpha B-crystallin (*CRYAB*), a member of the small heat shock protein family, is involved in apoptosis, inflammation, and redox regulation, and is associated with obesity and highly expressed in obese children (54). *CRYAB* expression was increased in the Over group in the present study. Scavenger receptor class A member 5 (*SCARA5*) is highly expressed in white adipose tissue and promotes adipocyte differentiation by regulating mesenchymal stem cells (MSCs) (55). *SCARA5* expression was also increased in the Over group in this study. Although direct evidence linking epiregulin (*EREG*), atonal bHLH transcription factor 8 (*ATOH8*), and terminal nucleotidyltransferase 5B (*TENT5B*) to adipogenesis or triglyceride accumulation is limited, these genes are associated with adipokine signaling, leptin regulation, or MSC function (56, 57). Their expression patterns in this study were consistent with these reports. Transmembrane (TMEM) proteins are embedded in various organelle membranes and play structural and functional roles in lipid droplet formation (24, 58). *TMEM160* is linked to human obesity and lipid metabolism in dairy cows (59). In the current study, *TMEM160* expression was also increased in the Over group. Our previous study showed that *TMEM273* expression was positively correlated with linoleic and arachidonic acid contents, and negatively correlated with oleic and palmitic acid contents in Dorper × Small-Tailed Han crossbred sheep (9). In the present study, *TMEM273* expression was also decreased with increased fat deposition of adipocytes. These results confirm that the reliability of the sequencing data and further demonstrate that *PDK4* positively regulates triglyceride deposition in ovine adipocytes.

Multi-omics analysis demonstrated that *TMEM273* exerted the strongest influence on other lipids or genes, and was closely correlated with 7 DELs (|correlation coefficient| >0.9), including TG(18:0_16:0_16:0) + NH_4_, TG(18:1_22:4_22:4) + NH_4_, TG(12:1e_6:0_20:3) + H, TG(36:1e) + Na, DG(18:0_18:2) + NH_4_, DG(18:0_18:1) + NH_4_, and PG(18:1_18:1) + NH_4_. *TMEM273* expression was increased in *PDK4*-knockout NIH/3 T3 cells but decreased during adipocyte differentiation. These findings suggest that *TMEM273* is negatively correlated with *PDK4* overexpression and fat deposition, and may be involved in lipid metabolism in sheep.

### Limitations of the study

This study has several limitations. A small number of biological replicates (*n* = 3–6) were used for sequencing. To confirm the reliability of RNA-seq data, 11 differentially expressed genes (DEGs) were selected by qPCR validation. The results showed that the expression levels of these genes were consistent with the RNA-seq data, and their expression patterns during adipocyte fat deposition were also consistent with those reported in other studies.

This study was conducted based on our previous findings regarding the correlation between *PDK4* and adipocytes (*in vitro*) as well as intramuscular fat (*in vivo*) (4, 5). Independent *in vivo* validation is lacking in the current study, and we will conduct relevant experiments in the next step. To verify the evolutionarily conserved functions of *PDK4*, ovine and murine adipocytes were used. Although ruminants and monogastric animals exhibit distinct lipid metabolism characteristics, the in vitro adipocyte lipogenesis are similar. Meanwhile, similar trends in fat deposition changes in ovine and murine adipocytes indicate evolutionarily conserved functions of *PDK4*.

Unfortunately, 13 DEGs without functional annotations were not investigated or discussed; experiments that confirm *TMEM273* is directly regulated by *PDK4* are missing; the underlying regulatory mechanisms differentially expressed lipids (DELs) and *TMEM273* remain insufficiently explored. These issues will be addressed gradually in future experiments. Despite these limitations, the current study still provides valuable information regarding *PDK4*-mediated regulation of fat deposition in sheep.

## Conclusion

*PDK4* positively regulates fat deposition in both ovine and murine adipocytes. *TMEM273* expression is negatively affected by *PDK4* and correlated with lipid metabolism in sheep and mice.

## Data Availability

The datasets presented in this study can be found in online repositories. The names of the repository/repositories and accession number(s) can be found at: https://www.ncbi.nlm.nih.gov/, PRJNA1061388 https://www.ncbi.nlm.nih.gov/, PRJNA1061467.

## Glossary

PDK4: Pyruvate dehydrogenase kinase 4
TG: Triglyceride
IMF: Intramuscular fat
PDC: Pyruvate dehydrogenase complex
MLD: Muscular longissimus dorsi
AT: Adipose tissue
LD: Lipid droplet
PPARG: Peroxisome proliferator activated receptor gamma
PC: Phosphatidylcholine
DG: Diglyceride
TMEM273: Transmembrane protein 273
BSCL2: BSCL2 lipid droplet biogenesis associated, seipin
MSC: Mesenchymal stem cell
TCA: Tricarboxylic acid cycle
STAT5: Signal transducer and activator of transcription 5A
KLF15: KLF transcription factor 15
FOXO1: Forkhead box O1
CIDEA: Cell death inducing DFFA like effector A
PPARGC1A: PPARG coactivator 1 alpha
MAM: Mitochondria-associated membrane
ER: Endoplasmic reticulum
IP3R1: Inositol 1,4,5-trisphosphate receptor 1
VDAC1: Voltage dependent anion channel 1
GRP75: Heat shock protein family A (Hsp70) member 9
GAPDH: Glyceraldehyde-3-phosphate dehydrogenase
TGM2: Transglutaminase 2
EGR3: Early growth response 3
AIF1L: Allograft inflammatory factor 1 like
ATOH8: Atonal bHLH transcription factor 8
SCARA5: Scavenger receptor class A member 5
CRYAB: Crystallin alpha B
TENT5B: Terminal nucleotidyltransferase 5B
TMEM160: Transmembrane protein 160
EREG: Epiregulin
PE: Phosphatidylethanolamine
LPG: Lysophosphatidylglycerol
LSM: Lysosphingomyelin
PS: Phosphatidylserine
FA: Fatty acid
SPH: Sphingosine
PG: Phosphatidylglycerol
Cer: Ceramide
SM: Sphingomyelin
VLDL: CD320 antigen
TMEM41B: Transmembrane protein 41B
