# Supplementary material for: PDK4 gene positively regulates fat deposition in ovine adipocytes
Source: Front Nutr. 2025 Dec 12;12:1706055. doi: 10.3389/fnut.2025.1706055 (PMC12742214; doi:10.3389/fnut.2025.1706055)
Supplement: Supplementary file 1 [file Image_1.pdf]

gRNA

Validation results

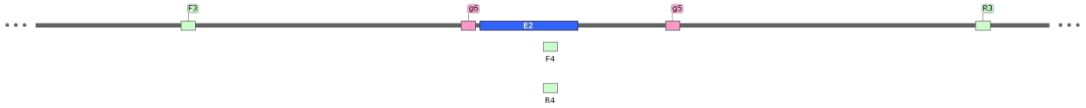

Clone No	Lane	Primer	Fragment	Result
#A8	1	F3/R3	754 bp	√+WT
WT	2	F3/R3	1169 bp	√
#A8	3	F3/R4	0 bp	√
WT	4	F3/R4	544 bp	√
#A8	5	F4/R3	0 bp	√
WT	6	F4/R3	646 bp	√

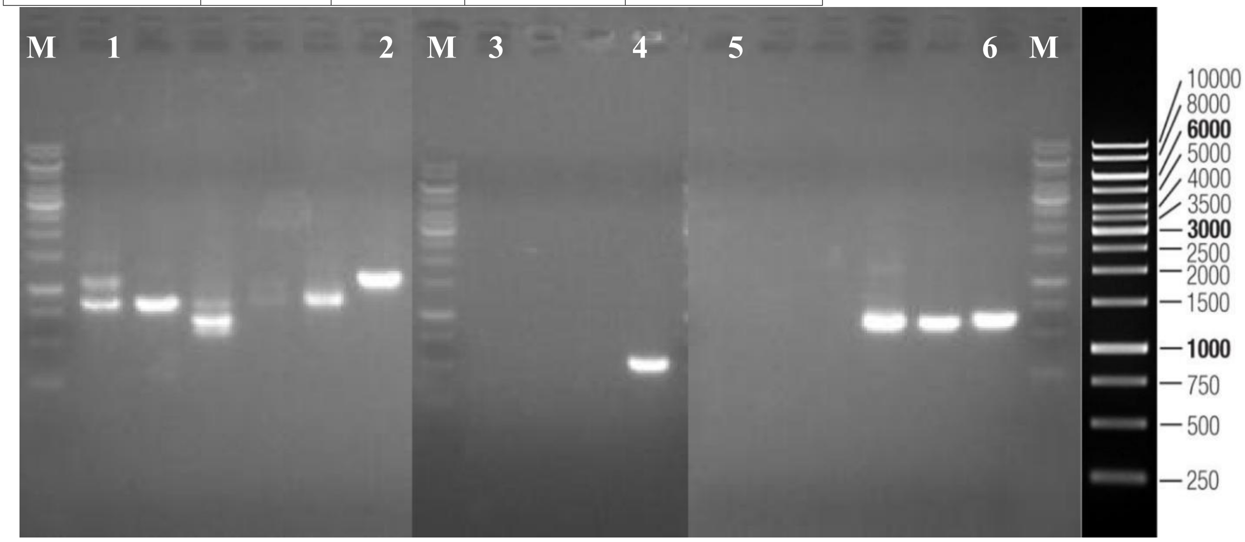

Sanger Sequencing

Compound heterozygous#A8:

Allele 1: del 308 bp

5'sequence:

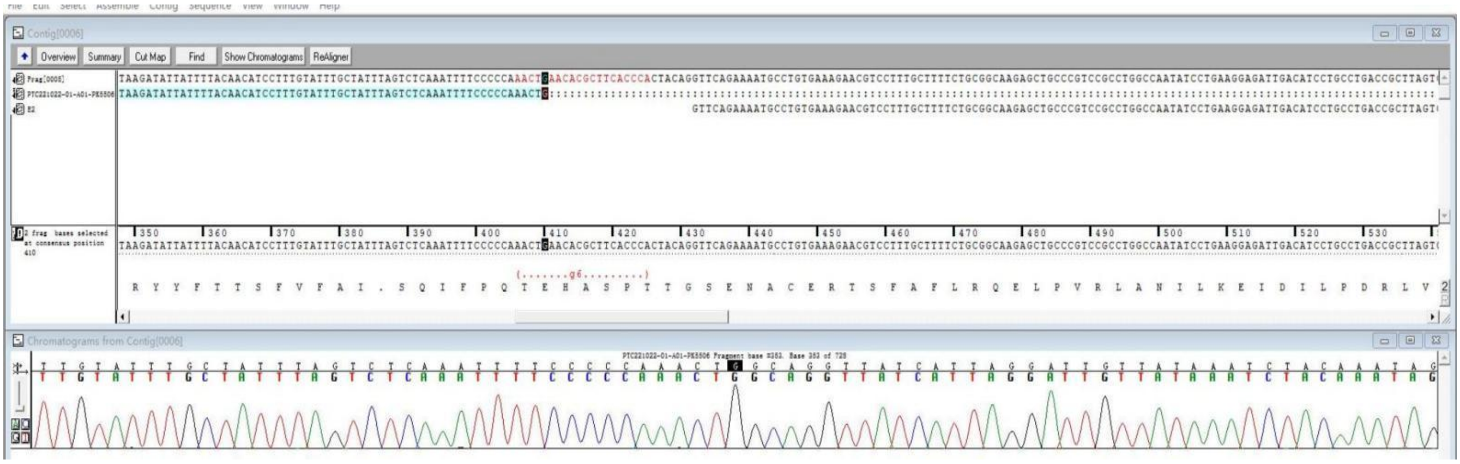

3'sequence:

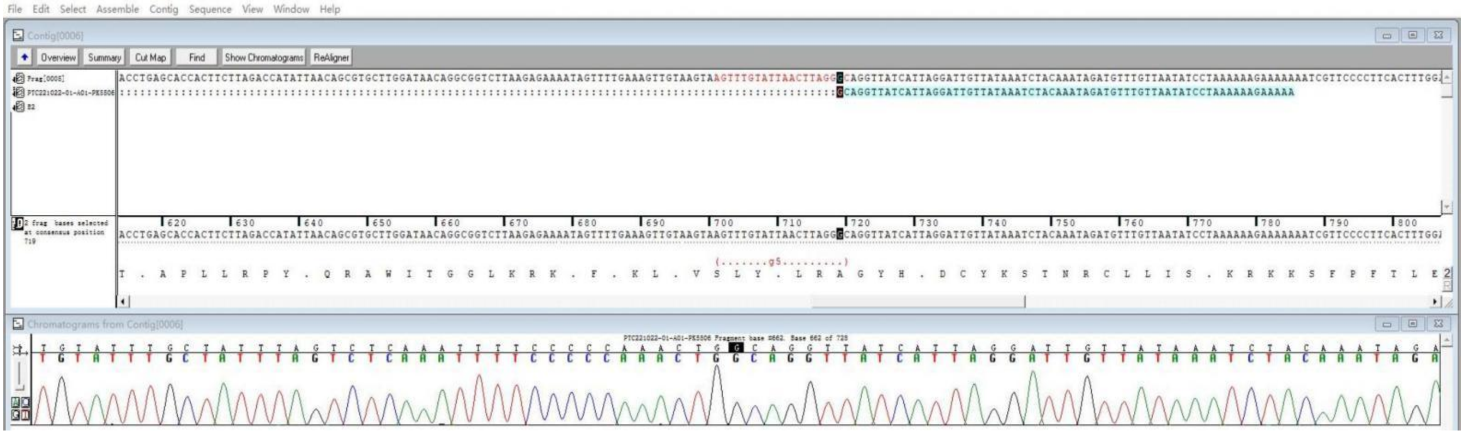

Allele 2: del 323 bp + knockin 313bp

5'sequence:

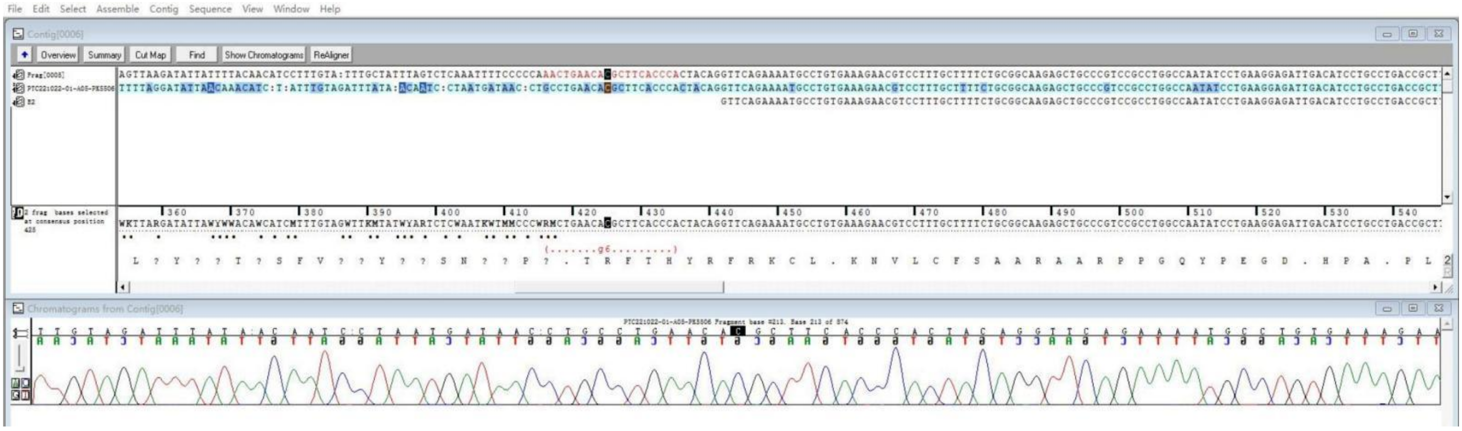

3’sequence:

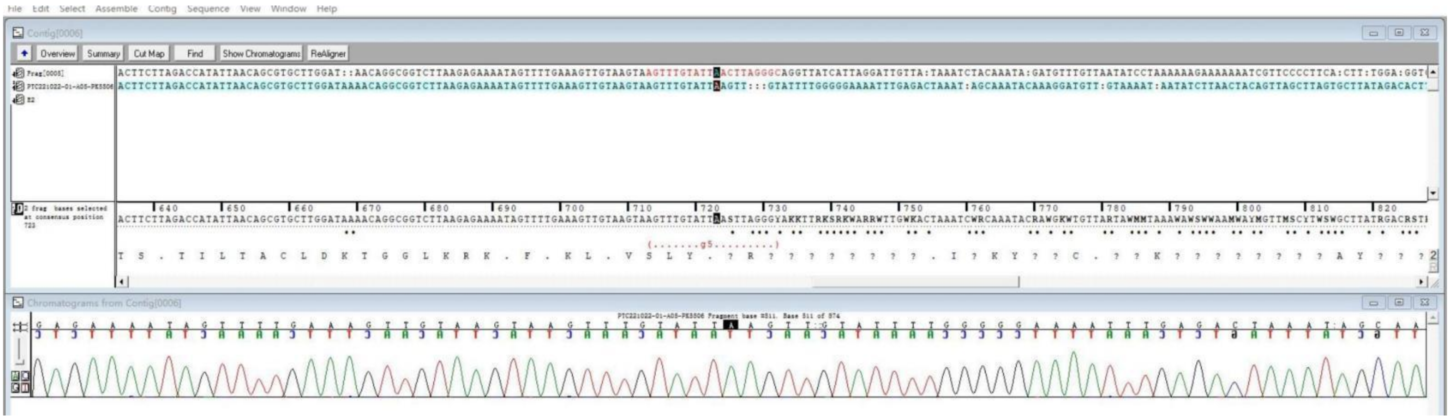

Allele 3: del 321 bp

5’sequence:

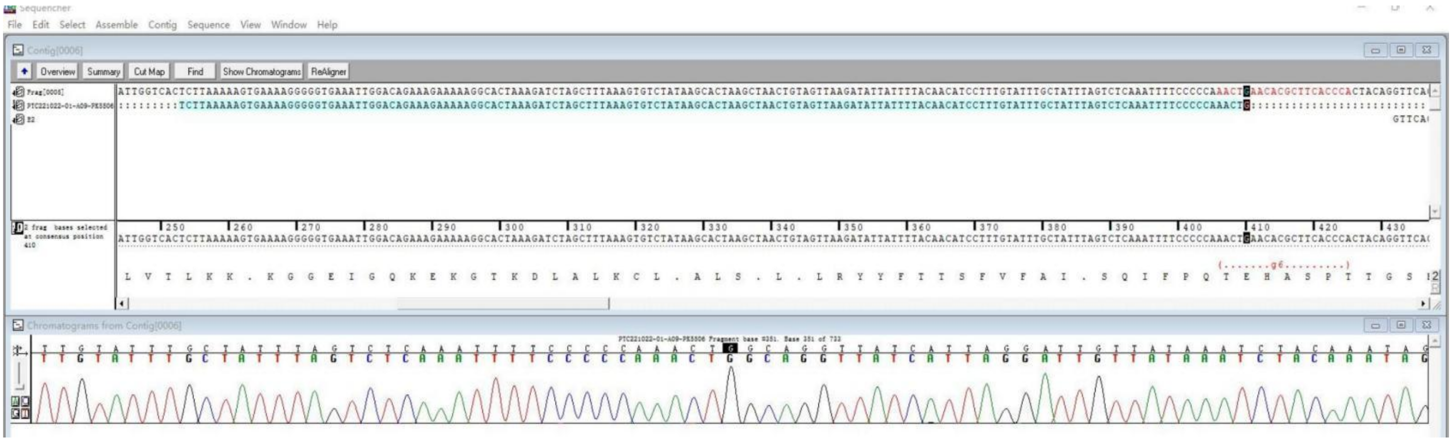

3’sequence:

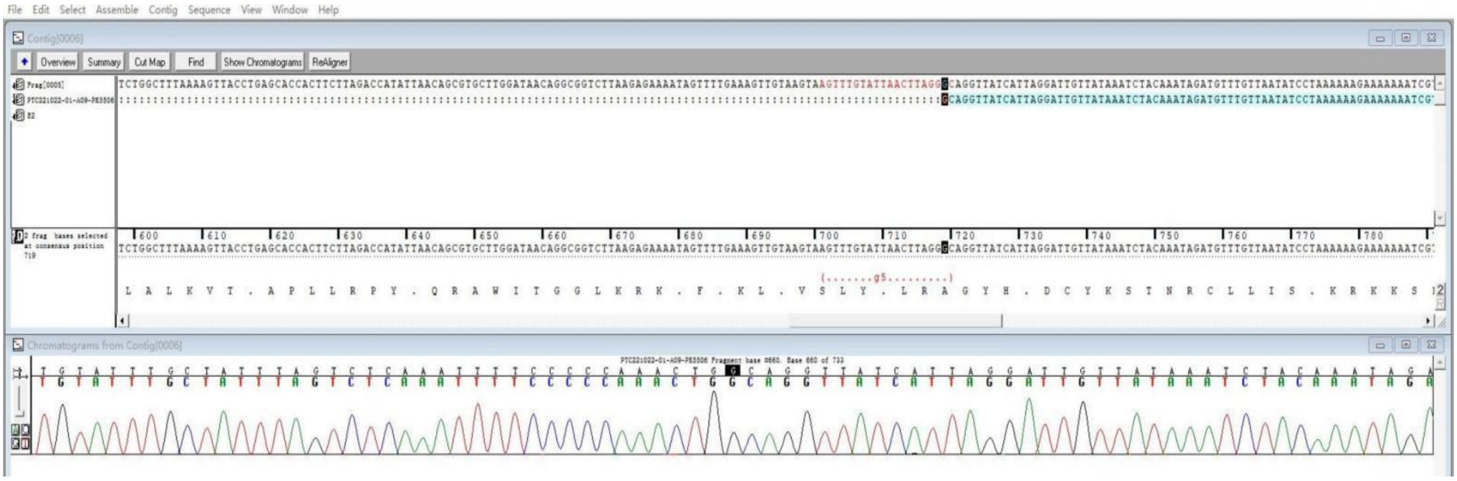

Allele 4: del 310 bp + knockin 313bp

5’sequence:

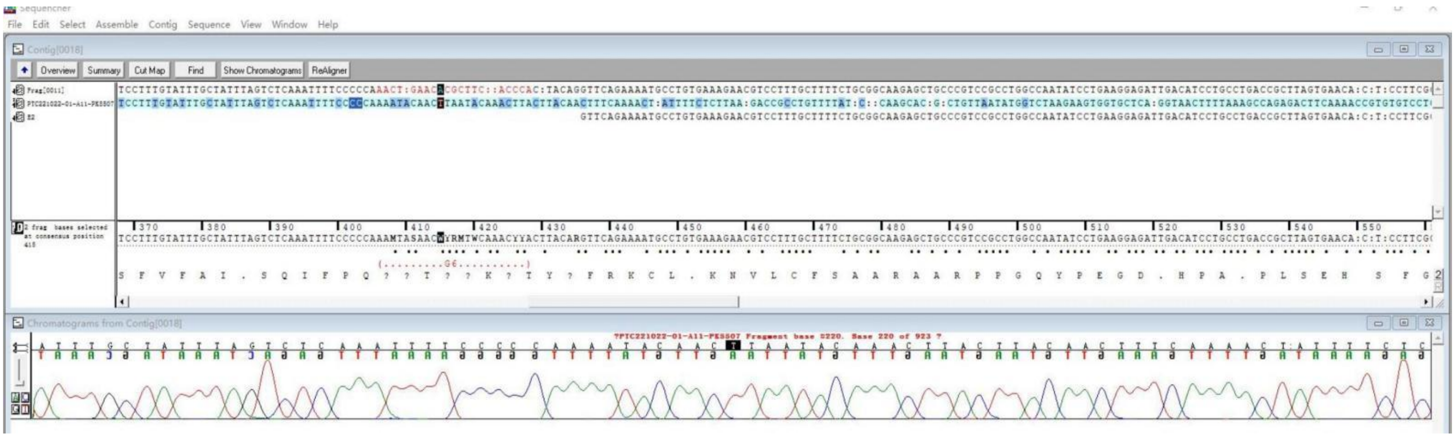

3’sequence:

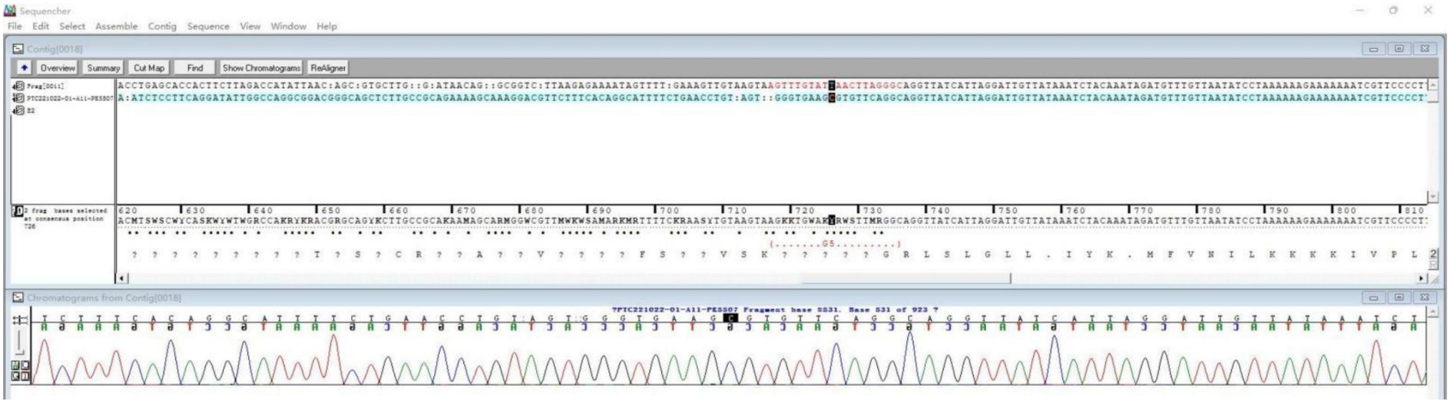

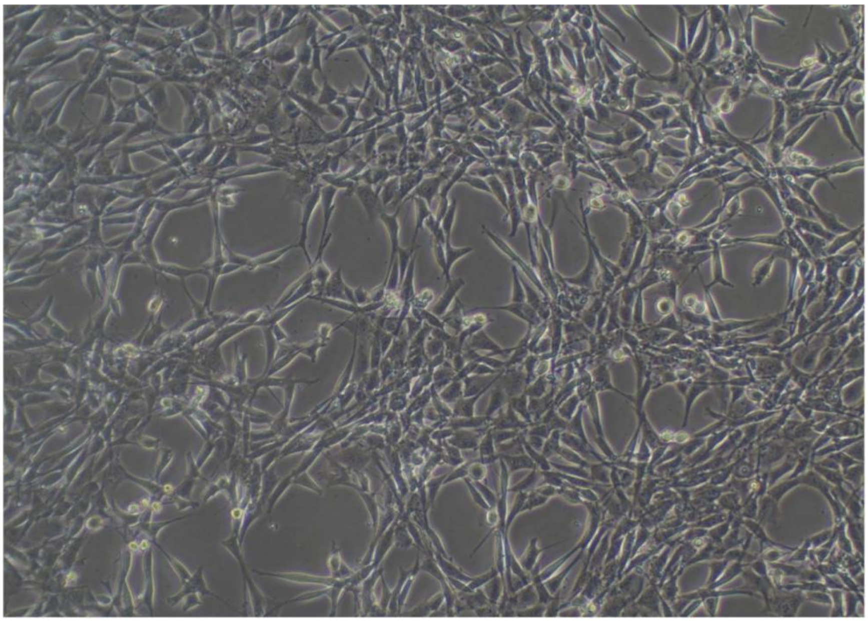

NIH/3T3-KO-Pdk4 100X

Figure1. Generation of *PDK4*-knockout NIH/3T3 Cell line
