# Supplementary material for: PDK4 gene positively regulates fat deposition in ovine adipocytes
Source: Front Nutr. 2025 Dec 12;12:1706055. doi: 10.3389/fnut.2025.1706055 (PMC12742214; doi:10.3389/fnut.2025.1706055)
Supplement: Supplementary file 2 [file Image_2.pdf]

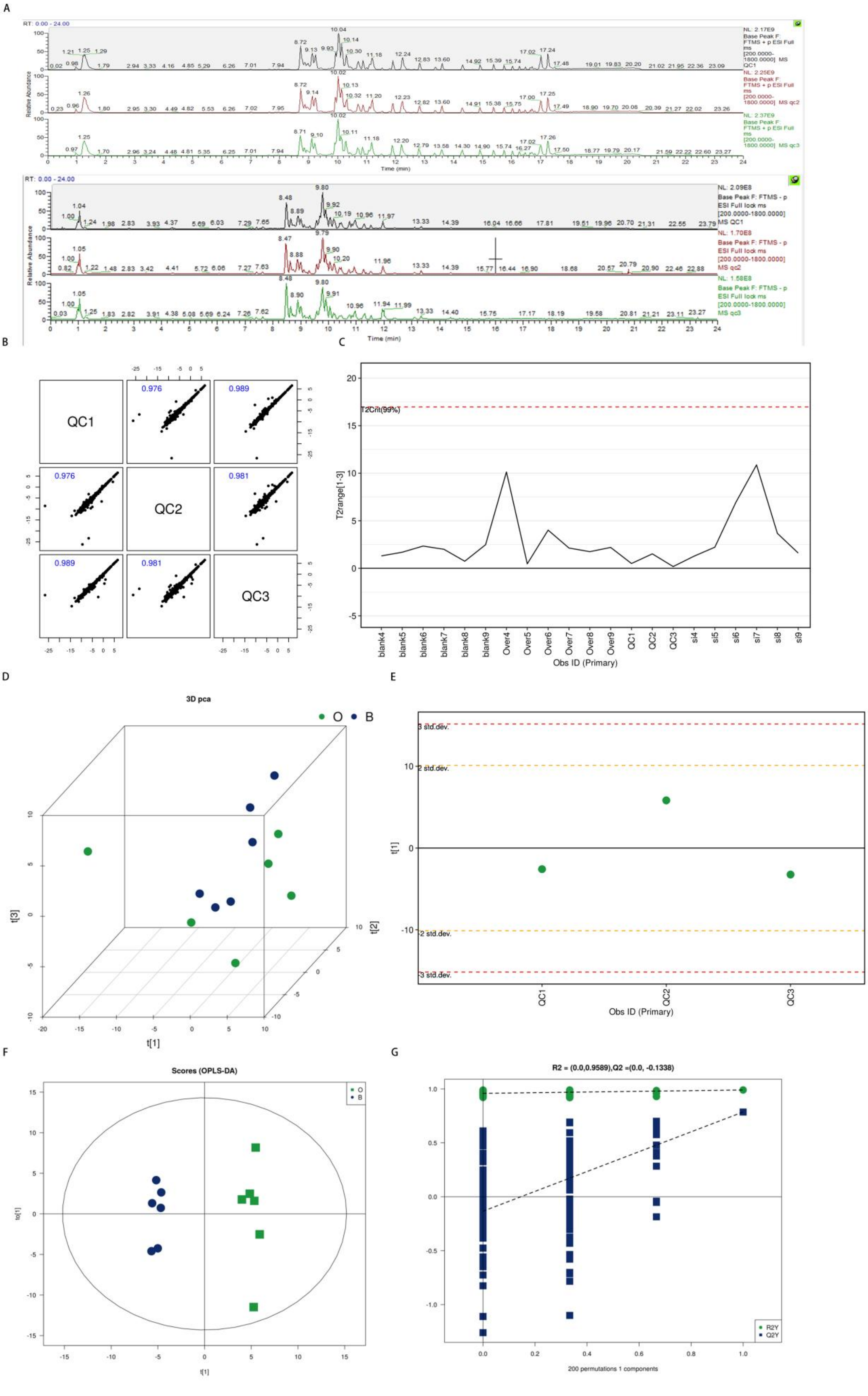

Figure2. Quality assessment of lipidomic data

A. Base peak chromatogram (BPC); similar peak patterns indicate good sample repeatability.

B. Pearson correlation analysis; correlation coefficients > 0.9 indicate good sample repeatability.

C. Hotelling's T^2 test; all samples fall within the 99% confidence interval, indicating good reproducibility.

D. Principal component analysis (PCA) of the overexpression (Over) and control groups.

E. Multivariate Control Chart (MCC); sample data within 3 units of deviation indicate high-quality results.

F. Orthogonal partial least squares-discriminant analysis (OPLS-DA) of the Over and control groups; clustered samples indicate good reproducibility.

G. OPLS-DA permutation test, a negative Q^2 value demonstrates that the model was reliable without overfitting
